# Colonization on Root Surface by a Phenanthrene-Degrading Endophytic Bacterium and Its Application for Reducing Plant Phenanthrene Contamination

**DOI:** 10.1371/journal.pone.0108249

**Published:** 2014-09-23

**Authors:** Juan Liu, Shuang Liu, Kai Sun, Yuehui Sheng, Yujun Gu, Yanzheng Gao

**Affiliations:** Institute of Organic Contaminant Control and Soil Remediation, College of Resources and Environmental Sciences, Nanjing Agricultural University, Nanjing, P.R. China; University Paris South, France

## Abstract

A phenanthrene-degrading endophytic bacterium, Pn2, was isolated from *Alopecurus aequalis* Sobol grown in soils contaminated with polycyclic aromatic hydrocarbons (PAHs). Based on morphology, physiological characteristics and the 16S rRNA gene sequence, it was identified as *Massilia* sp. Strain Pn2 could degrade more than 95% of the phenanthrene (150 mg·L^−1^) in a minimal salts medium (MSM) within 48 hours at an initial pH of 7.0 and a temperature of 30°C. Pn2 could grow well on the MSM plates with a series of other PAHs, including naphthalene, acenaphthene, anthracene and pyrene, and degrade them to different degrees. Pn2 could also colonize the root surface of ryegrass (*Lolium multiflorum* Lam), invade its internal root tissues and translocate into the plant shoot. When treated with the endophyte Pn2 under hydroponic growth conditions with 2 mg·L^−1^ of phenanthrene in the Hoagland solution, the phenanthrene concentrations in ryegrass roots and shoots were reduced by 54% and 57%, respectively, compared with the endophyte-free treatment. Strain Pn2 could be a novel and useful bacterial resource for eliminating plant PAH contamination in polluted environments by degrading the PAHs inside plants. Furthermore, we provide new perspectives on the control of the plant uptake of PAHs via endophytic bacteria.

## Introduction

Anthropogenic soil contamination has become a worldwide environmental problem in recent decades [1]. Organic pollutants present in soil can be taken up by plant roots and possibly followed by transportation along with the transpiration stream into shoots [2], which is a major pathway for toxic organic substances to reach the food chain [3]. Because plants form the basis of human and animal food chains, potentially harmful organic contaminants could find their way into human and animal populations via this route [4]–[7]. PAHs are a group of persistent organic contaminants commonly found in the soil environment [8]. Due to the potential mutagenic and/or carcinogenic properties of many PAHs [8], [9], an increased understanding of how to control plant uptake and the accumulation of PAHs from the environment could considerably benefit plant risk assessments [10].

Previous studies have shown that the uptake of PAHs by plants can be regulated and controlled through the addition of exogenous chemical reagents [11]–[13]. For instance, in the range of 0–296 mg·L^−1^, low concentrations (≤74.0 mg·L^−1^) of Brij35 (polyethylene glycol dodecyl ether, a nonionic surfactant) generally enhanced plant uptake and the accumulation of phenanthrene and pyrene, whereas Brij35 at relatively high concentrations (≥148 mg·L^−1^) markedly restricted the plant uptake of these PAHs [11]. In another study, at concentrations generally lower than 13.2 mg·L^−1^, Tween 80 (polyoxyethylenesorbitan monooleate, a nonionic surfactant) enhanced the plant uptake of phenanthrene and pyrene; however, when present at higher concentrations (13.2–105.6 mg·L^−1^), Tween 80 inhibited the uptake of both PAH compounds in the test plant [12]. However, compared with chemical surfactant-dependent technology, the biodegradation of PAHs inside plants is more environmentally friendly; therefore, the control of PAH accumulation in plants with endophytes has attracted considerable interest in recent years [14].

Endophytic bacteria, a type of endophyte, can be found in virtually every plant studied. Endophytic bacteria colonize the internal tissues of their host plants without causing any harm to host plants [15] and form a range of different relationships, including symbiotic, mutualistic, commensalistic and trophobiotic relationships [16]. Beneficial endophytic bacteria can promote plant growth, protect plants from external harsh environments and confer enhanced resistance to various pathogens by producing a range of natural products [17]. Furthermore, endophytic bacteria play an important role in soil fertility through phosphate solubilization, nitrogen fixation and other roles [17]. Additionally, some endophytic bacteria have the ability to degrade organic contaminants, showing potential applications for improving phytoremediation [16].

Endophytic bacteria possessing the capacity to degrade organic contaminants can be used to reduce organic pollution in soils and plants [16], [18]–[20]. For instance, the inoculation of plants with the poplar endophyte *Pseudomonas putida* VM1441 facilitated higher (40%) naphthalene degradation rates compared with endophyte-free plants in artificially contaminated soil [18]. In another study, the 2, 4-D-degrading endophytic bacterium *Pseudomonas putida* VM1450 could colonize the internal tissues of pea plants, maintain plant growth, and increase contaminant removal from the plants, demonstrating a 24–35% increase for these rates compared with the control [21]. Some endophytic bacteria can degrade organic pollutants *in vitro*, and others act inside the plant. Hence, utilizing endophytic bacteria to reduce the risk of organic contamination in plants would have immense advantages [21].

PAHs are among the most important environmental contaminants in China [22]. Agricultural soil with severe PAH contamination in the suburbs of industrial urban districts in China is primarily used for vegetable production [23]. In these PAH-contaminated sites, phenanthrene is always one of the dominant PAH compounds and can be taken up and accumulated in vegetables [8], [23]. However, to date, there have been no reports on using phenanthrene-degrading endophytic bacteria to remove phenanthrene from inside plants. Therefore, this study sought to isolate a phenanthrene-degrading endophytic bacterium and colonize it on the root surface of a plant. After inoculation, the cell counts of strain Pn2 and phenanthrene concentrations inside the plant were investigated to confirm whether the colonization of this endophyte could reduce plant phenanthrene contamination. In so doing, we provided a novel and useful bacterial resource for eliminating plant PAH contamination by degrading the PAHs inside plants. The results of this work are expected to benefit agricultural production, food safety, and human health linked to PAH-contaminated sites.

## Materials and Methods

### 2.1 Chemicals and media

The phenanthrene used (>99%, MW 178.2 g·mol^−1^, Fluka Germany) had a pure water solubility of 1.2 mg·L^−1^ and a log *K_oc_* of 4.15 [24]. All solvents used were pure analytical grade. A highly concentrated stock solution of phenanthrene (10.0 g·L^−1^ in acetone) was prepared.

The mineral salt medium (MSM) [25] contained 1.50 g·L^−1^ (NH_4_)_2_SO_4_, 1.91 g·L^−1^ K_2_HPO_4_·3H_2_O, 0.50 g·L^−1^ KH_2_PO_4_, 0.20 g·L^−1^ MgSO_4_·7H_2_O, and 1 mL trace element solution (0.1 mg·L^−1^ CoCl_2_·6H_2_O, 0.425 mg·L^−1^ MnCl_2_·4H_2_O, 0.05 mg·L^−1^ ZnCl_2_, 0.01 mg·L^−1^ NiCl_2_·6H_2_O, 0.015 mg·L^−1^ CuSO_4_·5H_2_O, 0.01 mg·L^−1^ Na_2_MoO_4_·2H_2_O, 0.01 mg·L^−1^ Na_2_SeO_4_·2H_2_O). Solid medium plates were prepared by adding 16 g·L^−1^ agar into the above liquid medium.

The PMM media was MSM supplemented with phenanthrene at concentrations as required (50–200 mg·L^−1^). For the liquid PMM medium, after phenanthrene (10.0 g·L^−1^ in acetone) was added into the sterile flasks, the acetone was volatilized with sterile air followed by the addition of the MSM. For the solid PMM medium plates, after phenanthrene (10.0 g·L^−1^ in acetone) was mixed with the melting MSM, the medium was poured into the plates and the acetone was volatilized with sterile air. The naphthalene, acenaphthene, anthracene, pyrene and benzo(a)pyrene were added into the MSM in the same manner with phenanthrene.

The 1/10 Luria-Bertani (LB) was a 1/10 dilution of LB with MSM. Solid medium plates were prepared by adding 16 g·L^−1^ agar to the liquid medium.

In artificially contaminated Hoagland nutrient solution [26], phenanthrene (10.0 g·L^−1^ in acetone) was added into the solution (as in the PMM).

### 2.2 The isolation and identification of the phenanthrene-degrading endophytic bacterium

*Alopecurus aequalis* Sobol, which is common in China and is also the dominant plant in PAH-contaminated fields, was collected from a petrochemical factory in Nanjing for the isolation of phenanthrene-degrading endophytic bacteria (permission was obtained from the owner of this private land to perform the study on this site). The total PAH concentration in the naturally contaminated soil was 178 mg·kg^−1^ dry weight of soil, and the phenanthrene concentration was 8.62 mg·kg^−1^ dry weight of soil [27]. Plant samples were surface-disinfected to remove epiphytes, as described previously [14]. The epiphytic bacteria were detected by pressing the plants onto fresh LB agar plates to confirm that the surface disinfection process was successful.

Plant materials (0.2 g, fresh weight) were ground with a pestle in a mortar that contained 10 mL sterile distilled water. Serial dilutions were prepared and put into 250-mL flasks containing 100 mL PMM (50 to 200 mg·L^−1^ of phenanthrene). The enrichment procedure was performed with phenanthrene as the sole carbon source, as described previously [25]. The final enrichment culture was spread on PMM plates (150 mg·L^−1^ of phenanthrene). The bacterial colonies producing a clear zone were selected as the phenanthrene-degrading bacterial strains. After purification, the identification of the phenanthrene-degrading bacteria was performed as previously described [28], and these bacterial colonies were determined to be the same strain, Pn2.

For phenanthrene residue measurements, the same volume of methanol was added into the flasks to solve residual phenanthrene; the mixture solution was ultrasonicated for 30 min and then filtered with 0.22- µm filters (polytetrafluoroethylene, PTFE). Phenanthrene residue in the flask was measured using a high-performance liquid chromatograph (HPLC, Shimadzu LC-20AT, Japan) fitted with a 150-mm×4.6-mm reverse-phase C_18_ column using methanol and water (90∶10, v: v) in the mobile phase at a flow rate of 0.9 mL·min^−1^. Chromatography was performed at 40°C. Phenanthrene was detected at 245 nm, the injection volume was 20 µL, and its limit of detection was 2.2 µg·L^−1^. The biodegradation rate was calculated by comparing the peak area with that of a phenanthrene standard according to the phenanthrene standard curve.

### 2.3 Degradation of PAHs by strain Pn2

An inoculum of strain Pn2 was prepared by cultivating bacteria in 1/10 LB medium for 48 h at 30°C and 180 r·min^−1^ in a rotary shaker. The cells were collected via centrifugation at 8,000 r·min^−1^ for 10 min, washed twice with MSM, and resuspended at 2.0−3.2×10^8^ colony-forming units (cfu·mL^−1^).

The degradation dynamics of the phenanthrene and the growth curve of strain Pn2 were determined. The cells were inoculated at the 5% (v/v) level into a 50 mL flask containing 20 mL PMM supplemented with 150 mg·L^−1^ of phenanthrene (pH 7.0) and incubated at 30°C and 180 r·min^−1^ in a rotary shaker. Media containing inactivated Pn2 served as a control (the Pn2 cells were inactivated through moist heat sterilization after collected via centrifugation and then washed twice with MSM). Samples of culture medium were periodically withdrawn, the phenanthrene residue was measured using HPLC as described above, and the cell counts in the flasks were estimated via plate counting.

The tolerance of strain Pn2 to each type of PAH was investigated by inoculating Pn2 on MSM plates containing one of the following PAHs: naphthalene (200 mg·L^−1^), acenaphthene (200 mg·L^−1^), anthracene (100 mg·L^−1^), pyrene (50 mg·L^−1^), or benzo(a)pyrene (10 mg·L^−1^). The plates were incubated for 3–7 days at 30°C, and bacterial growth was monitored regularly. The ability of strain Pn2 to degrade these PAHs was tested as previously described [25], [29]–[32].

All experiments were performed in triplicate.

### 2.4 Greenhouse container experiments

To trace the distribution of inoculated bacteria colonizing in the inner plant, Pn2 was labeled with antibiotic resistance. The antibiotic resistance of strain Pn2 was tested on 1/10 LB solid medium plates containing gentamicin, ampicillin, kanamycin, chloramphenicol, streptomycin, rifampicin or tetracycline. Each antibiotic was added aseptically to the medium at 0, 10, 20, 50, 75, and 100 mg·L^−1^. The cultures were incubated at 30°C for 3 days.

Bacterial strain Pn2 was inoculated in 1/10 LB medium at 30°C 180 r·min^−1^ until OD*_600nm_* = 0.55. The bacterial cells were collected via centrifugation and washed twice with the same volume of MSM. Ryegrass was chosen as the experimental plant because of its rapid growth, well-developed root system, and tolerance of high levels of PAHs [33]. The container experiment consisted of two levels of phenanthrene contamination (0 and 2 mg·L^−1^ Hoagland solution) and five treatments: Uncontaminated solution + ryegrass (UR), uncontaminated solution + ryegrass + bacteria (URB), contaminated solution (CK), contaminated solution + ryegrass (CR) and contaminated solution + ryegrass + bacteria (CRB). All treatments were replicated three times.

The ryegrass seeds were disinfected with 75% ethanol for 5 min and then placed in a 30°C incubator to germinate for 48 h. After that, the seedlings of ryegrass were grown in nursery cups until the plants were approximately 10-cm tall with relatively mature roots, at which point they were used for the contamination experiments. Ryegrass roots were soaked in MSM with Pn2 or inactivated Pn2 (OD*_600nm_* = 0.55) for 3 hours and then replanted in a 300-mL brown glass volumetric flask containing 250 mL of Hoagland solution (phenanthrene: 0 or 2 mg·L^−1^, 10 plants in each bottle). Plant cultivation was carried out in environmental growth chambers set at 25°C under a 12 h light/12 h dark cycle for 12 days.

After 12 days of inoculation, the plants were harvested and rinsed three times with sterile distilled water. After drying the plant surface with filter paper, the fresh weight, height and root length of the plant samples were determined. The fresh plant samples were freeze-dried for 72 h and then reweighed to determine their dry weight. After that, the root and stem materials (0.2 g fresh weight) were surface-sterilized, ground in a mortar and then diluted for the cell counting of strain Pn2 on MSM medium plates supplemented with 75 mg·L^−1^ ampicillin, 25 mg·L^−1^ chloramphenicol and 150 mg·L^−1^ phenanthrene. The cell numbers of strain Pn2 colonizing the ryegrass root surfaces [34] and those released into the Hoagland solution were counted with the plate counting method. The bacterial colonies grown on the plates were randomly selected to perform 16S rRNA gene sequencing, and the phenanthrene-degrading ability of the isolated bacterial strains was also investigated to ensure that these isolates were strain Pn2.

The phenanthrene contents in the Hoagland solution and inside the plants after treatment were measured. The plant samples were freeze-dried for 72 h, crushed, homogenized and analyzed for residual phenanthrene via HPLC [35]. Plant samples were extracted by ultrasonication for 30 min in a 1∶1 (v/v) solution of acetone and dichloromethane (DCM) with anhydrous Na_2_SO_4_ to remove moisture, followed by centrifugation. This process was repeated 3 times. Then, the supernatant was filtered through a column with 2 g of silica gel containing a 10 mL 1∶1 (v/v) elution of DCM and hexane. The solvent fractions were evaporated and exchanged with methanol to a final volume of 10 mL. The extracts were analyzed by HPLC as described above. The recoveries of phenanthrene investigated in the plant samples averaged ≥95% (n = 5, RSD ≤2.52%) for phenanthrene after the entire procedure. Regarding the water samples, 10 mL of water solution containing phenanthrene was mixed with 10 mL of methanol; the mixture solution was ultrasonicated for 30 min and then filtered through a 0.22 µm filter (PTFE) prior to analysis using HPLC.

### 2.5 Statistical analysis

All data were analyzed using Microsoft Office Excel 2003. Each data point in the figures and tables is an average value, and the standard deviation from three parallel samples is shown in the figures as an error bar. The differences in Pn2 cell number colonized in plant in the contaminated and uncontaminated solutions, as well as plant biomass, phenanthrene concentration and accumulation in plant across the treatments, were also analyzed via one-way ANOVA using SPSS version 11.0 (SPSS, Inc.) with a confidence limit of 95%.

## Results

### 3.1 Isolation and identification of strain Pn2

A phenanthrene-degrading endophytic bacterium, Pn2, was isolated from the root interiors of *Alopecurus aequalis* Sobol grown at a PAH-contaminated site.

**Figure 1:**
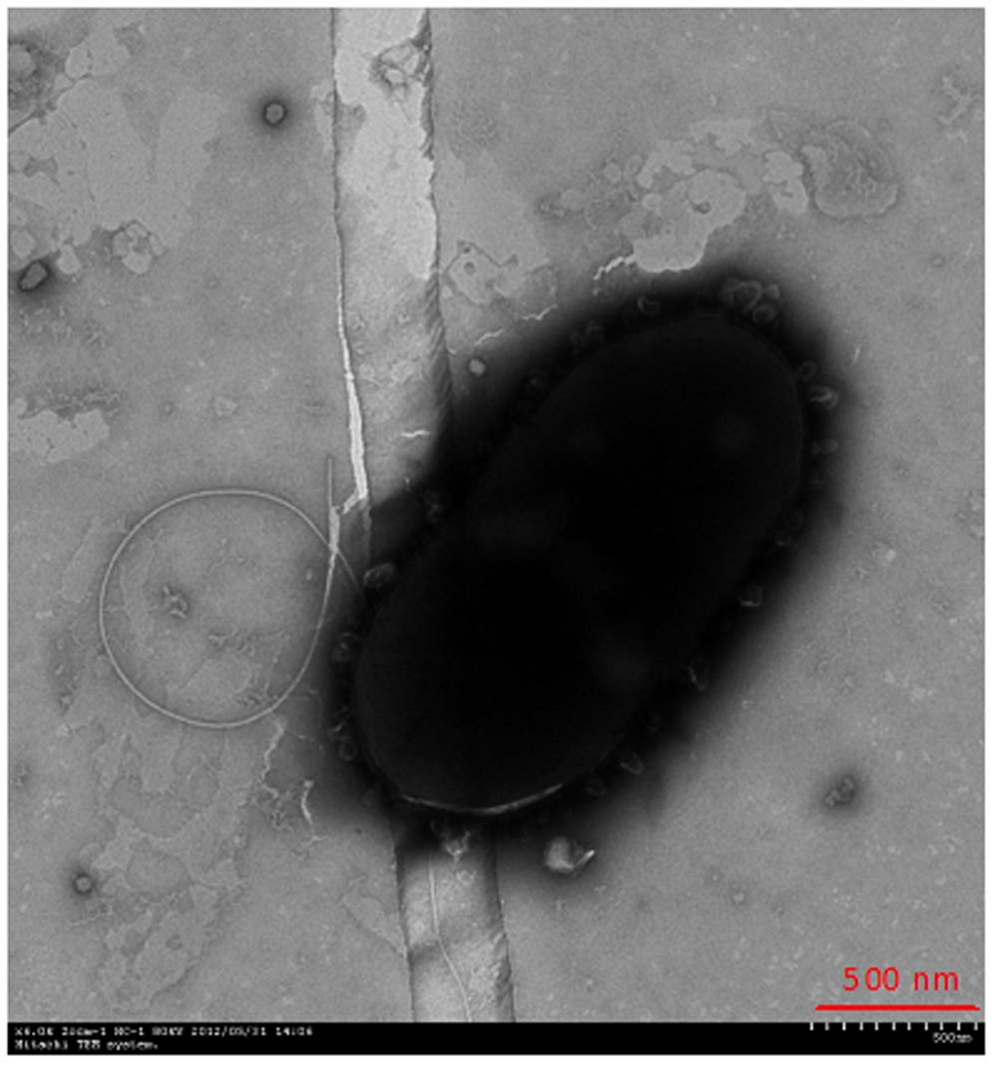
Transmission electron micrograph of strain Pn2 (×6.0K Zoom-1 HC-1 80 kV).

The taxonomic properties of strain Pn2 were as follows: a short, rod-shaped, gram-negative bacterium with flagellum (Fig 1) that is positive for oxidase, catalase and urease; negative for benzene alanine deaminase and β-galactosidase; nitrate reduction-negative; negative for gelatin hydrolysis; positive for esculin hydrolysis; negative for glucose, lactose and fructose hydrolysis; positive for the assimilation of glucose, arabinose, mannose, maltose and starch; negative for the assimilation of citric acid, malic acid and adipic acid; negative on the Voges-Proskauer test; and negative on the methyl red test.

The GenBank accession number of the 16S rRNA gene sequence of strain Pn2 is JX270637. The 16S rRNA gene sequence was compared with available sequences in the GenBank. The result showed that it was 98% identical to that of the 16S rRNA gene of *Massilia niastensis* 5516S-1T (GenBank Accession No. EU808005). Based on these characteristics, the isolate Pn2 was preliminarily identified as *Massilia* sp.

### 3.2 Degradation of PAHs by strain Pn2

#### 3.2.1. Degradation dynamics of phenanthrene by strain Pn2

**Figure 2:**
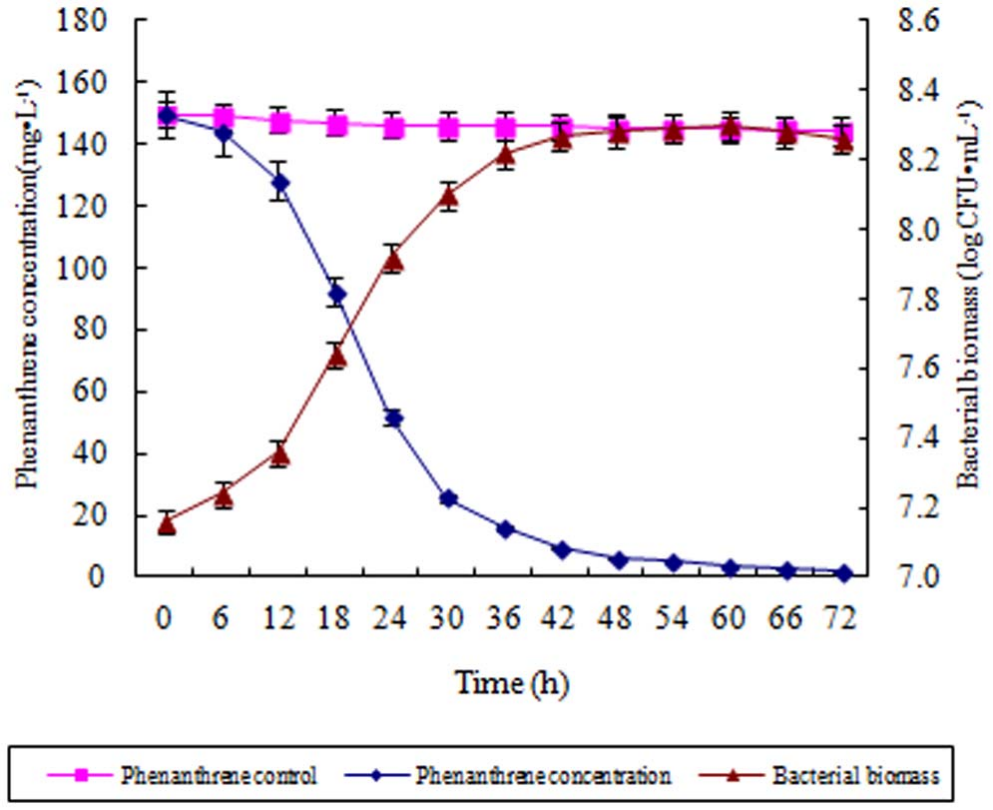
The degradation dynamics of phenanthrene and growth curve of strain Pn2 with phenanthrene as its sole carbon source. Error bars are ± standard deviation (n = 3).

To investigate the degradation ability of phenanthrene by strain Pn2, the degradation dynamics were studied in liquid culture medium with phenanthrene as the sole carbon source. The time course studies for the degradation of phenanthrene showed a rapid degradation by Pn2, with more than 80% of 150 mg·L^−1^ phenanthrene degraded within 30 hours (Fig 2). At the same time, rapid growth was observed for strain Pn2, the cell counts for strain Pn2 quickly increased during the first 30 hours. After inoculation for 36 hours, the cell counts of strain Pn2 increased very slowly. Correspondingly, the degradation of phenanthrene by Pn2 after 36 hours also slowed.

#### 3.2.2. Tolerance and degradation ability of strain Pn2 for each PAH type

Strain Pn2 grew well on MSM plates with naphthalene (200 mg·L^−1^), acenaphthene (200 mg·L^−1^), phenanthrene (150 mg·L^−1^), anthracene (100 mg·L^−1^), pyrene (50 mg·L^−1^), and benzo(a)pyrene (10 mg·L^−1^), respectively. The degradation ability of strain Pn2 for each type of PAH is shown in Table 1. Pn2 could degrade more than 95% of naphthalene (100 mg·L^−1^) or acenaphthene (100 mg·L^−1^) within 2 days. When anthracene (50 mg·L^−1^) or pyrene (20 mg·L^−1^) was used as the substrate, the degradation rate decreased markedly. Only 28% of anthracene and 68% of pyrene was degraded by strain Pn2 after incubation for 3 days and 14 days, respectively. However, when benzo(a)pyrene (10 mg·L^−1^) was used as the substrate, the biodegradation was almost completely restrained. In summary, heavy weight PAHs were less degraded by Pn2, the reason may be due to the lower capability of Pn2 to metabolize them, as well as the really low solubility of heavy weight PAHs in water, making them not accessible for degradation.

**Table 1:** The degradation ability of strain Pn2 for each PAH type.

PAH	Initial concentration (mg·L^−1^)	Degrading time (day)	PAH degradation (%)	Bacterial biomass (log CFU·mL^−1^)
Control	0	0	ND	6.94±0.09
Naphthalene	100	1	73.62±2.96	8.07±0.05
		2	95.81±3.57	8.21±0.05
Acenaphthene	100	1	27.04±2.30	7.49±0.04
		2	97.28±1.72	8.30±0.01
Anthracene	50	1.5	20.86±1.55	7.24±0.14
		3	27.81±6.74	7.35±0.06
Phenanthrene	50	1.5	93.62±6.96	8.04±0.02
		3	99.57±0.38	8.13±0.04
Pyrene	20	7	9.64±1.34	7.12±0.06
		14	67.59±1.75	7.56±0.11
Benzo(a)pyrene	10	7	1.20±0.83	7.04±0.05
		14	2.51±0.58	7.09±0.03

ND means not detected; Error bars are ± standard deviation (n = 3).

### 3.3 Reducing the plant phenanthrene contamination with Pn2

#### 3.3.1. Antibiotic resistance of strain Pn2

To determine the survival rate of the inoculated bacteria colonizing the plant tissues, the phenanthrene-degrading endophytic isolate Pn2 was tested for antibiotic resistance. The results showed that strain Pn2 was resistant to ampicillin (≤75 mg·L^−1^) and chloramphenicol (≤25 mg·L^−1^) but not to the other antibiotics tested.

#### 3.3.2. Cell counts of strain Pn2 colonizing ryegrass

In greenhouse container experiments, most bacterial cells of strain Pn2 colonized the root surfaces of ryegrass, and some of them could be released into the Hoagland solution. With the decrease of phenanthrene in the solution from day 6 to day 12, the cell counts on the root surfaces and in the solution decreased as well (Table 2). The cell counts of strain Pn2 in the contaminated solution were significantly (p<0.05) higher than those in the uncontaminated solution both on the root surfaces and in the Hoagland solution.

**Table 2:** Plate counts of strain Pn2 colonizing in plant tissues and in Hoagland solution after inoculation for 6 days and 12 days.

Different treatments	Cell counts after 6 days				Cell counts after 12 days			
	Shoot (×10^5^ CFU·g^−1^ FW)	Root (×10^5^ CFU·g^−1^ FW)	Root surface (×10^5^ CFU·g^−1^ FW)	Solution (×10^5^ CFU·mL^−1^)	Shoot (×10^5^ CFU·g^−1^ FW)	Root (×10^5^ CFU·g^−1^ FW)	Root surface (×10^5^ CFU·g^−1^ FW)	Solution (×10^5^ CFU·mL^−1^)
URB	0.25±0.03	0.72±0.09	82.15±4.40	3.42±0.37	0.14±0.04	0.31±0.06	48.02±4.67	0.08±0.02
CRB	1.32±0.19*	2.87±0.38*	181.13±8.77*	5.75±0.46*	0.79±0.08*	1.64±0.28*	81.37±6.31*	0.96±0.11*

URB (planted ryegrass and inoculated with strain Pn2 in uncontaminated solution), CRB (planted ryegrass and inoculated with strain Pn2 in contaminated solution). Error bars are ± standard deviation (n = 3). * indicates a significant difference between contaminated and uncontaminated solutions (P<0.05).

The Pn2 cells that colonized the root surface could penetrate into the ryegrass tissues; they demonstrated good survival during the experiment period. During the decrease of phenanthrene in the solution from day 6 to day 12, the cell counts in the plant roots and shoots both decreased (Table 2). The cell counts of strain Pn2 in the ryegrass tissues in the contaminated solution were also significantly (p<0.05) higher than those in the uncontaminated solution.

#### 3.3.3. Strain Pn2 promotion of ryegrass growth

After 12 days of incubation with phenanthrene, ryegrass growth did not exhibit significant stress or toxic effects in the contaminated solution (Table 3). Furthermore, the colonization of ryegrass by strain Pn2 significantly increased the ryegrass fresh weight and dry weight (p<0.05). There were also significant differences between each treatment in the height and root length of the ryegrass. Compared with the endophyte-free treatment, the ryegrass height and root length were both significantly higher in the endophyte-inoculated treatments (p<0.05).

**Table 3:** Root and shoot biomass of ryegrass after inoculation for 12 days.

Different treatments	Root			Shoot		
	Length (mm)	Fresh weight (mg·pot^−1^)	Dry weight (mg·pot^−1^)	Length (mm)	Fresh weight (mg·pot^−1^)	Dry weight (mg·pot^−1^)
UR	72.33±5.56	95.35±11.27	10.85±1.52	123.19±4.44	196.27±17.67	16.51±1.11
URB	84.67±3.28*	156.11±11.99*	16.97±1.82*	145.13±4.94*	289.42±23.00*	25.21±2.17*
CR	69.67±4.36	101.94±9.83	11.32±1.46	126.34±4.19	201.00±17.42	17.25±2.31
CRB	81.52±4.10*	162.66±11.55*	17.13±2.18*	141.62±3.99*	325.40±24.15*	28.41±2.25*

UR (planted ryegrass in uncontaminated solution), URB (planted ryegrass and inoculated with strain Pn2 in uncontaminated solution), CR (planted ryegrass in contaminated solution), CRB (planted ryegrass and inoculated with strain Pn2 in contaminated solution). Error bars are ± standard deviation (n = 3). * indicates a significant difference between endophyte-inoculated and endophyte-free treatments (P<0.05).

#### 3.3.4. Reducing phenanthrene concentrations in ryegrass via strain Pn2

PAHs could be taken up by the plants, transported from the roots to the shoots with the transpiration stream and metabolized in the plants [10], [12]. The uptake of phenanthrene from the solution by the ryegrass roots and shoots after 12 days is shown in Table 4. The concentrations of phenanthrene in the ryegrass roots and shoots with Pn2 inoculation were significantly lower than those in the treatments without Pn2 inoculation (p<0.05). The phenanthrene accumulation in roots and shoots also significantly decreased with Pn2 inoculation (p<0.05). At the end of the greenhouse container experiments, the phenanthrene concentration in the contaminated solution without the plants was 1.85 mg·L^−1^, whereas the phenanthrene concentration with the plants was only 0.36 mg·L^−1^, indicating that most of the phenanthrene in the solution was taken up and metabolized by the plants.

**Table 4:** The concentration and plant concentration factors of phenanthrene in Hoagland solution and in ryegrass added with 2 mg·L^−1^ of phenanthrene in the solution.

Different treatments	The concentration of phenanthrene			Plant concentration factors (PCF)		Accumulation		Translocation factor (TF)
	Root (mg·kg^−1^)	Shoot (mg·kg^−1^)	Solution (mg·l^−1^)	Root (C_p_/C_s_)	Shoot (C_p_/C_s_)	Root (µg·pot^−1^)	Shoot (µg·pot^−1^)	SCF/RCF
CK	ND	ND	1.85±0.09	-	-	-	-	-
CR	35.42±2.71	12.76±1.22	0.36±0.05	98.38	35.44	0.40±0.02	0.22±0.03	0.36
CRB	16.25±1.30*	5.47±0.47*	0.22±0.04*	73.86	24.86	0.28±0.03*	0.15±0.01*	0.33

CK (contaminated solution), CR (planted ryegrass in contaminated solution), CRB (planted ryegrass and inoculated with strain Pn2 in contaminated solution). PCF (Plant concentration factor, which is calculated as pollutant content in the plant roots or shoots (C_p_, mg·kg^−1^)/the concentration of pollutant in solution (C_s_, mg·l^−1^), PCF = C_p_/C_s_), TF (Plant tanslocation factor, that is shoot concentration factor (SCF)/root concentration factor (RCF), TF =  SCF/RCF). Error bars are ± standard deviation (n = 3). ND means not detected. * indicates a significant difference between endophyte-inoculated and endophyte-free treatments in contaminated solutions (P<0.05).

The plant concentration factor (PCF) describes the capability of plants to accumulate contaminants from direct contact with the environment, which in this study was calculated as the ratio of the phenanthrene concentration in the plant roots and shoots (*C_p_*) to that in the solution (*C_s_*), PCF = *C_p_*/*C_s_*. The plant translocation factor (TF) is defined as the ratio of the phenanthrene concentration in the shoot concentration factor (SCF) to that in the root concentration factor (RCF), TF =  SCF/RCF. A larger TF value indicates that more phenanthrene is translocated by ryegrass from the roots to the shoots. The PCF and TF of phenanthrene in ryegrass with Pn2 inoculation (24.86–73.86, 0.33) were lower than those in the no-inoculation treatment (35.44–98.38, 0.36) at 12 days.

## Discussion

Utilizing endophytic bacteria to reduce the organic contaminants inside plants could have considerable benefits in lowering the risk of plant organic contamination [14]. It was reported that the strain *Burkholderia fungorum* DBT1, isolated from sediment and capable of degrading PAH compounds such as naphtalene, phenanthrene and dibenzothiphene, was able to colonize internal roots of poplar and increase the plant phytodegradation capability once grown in presence of the above mentioned PAHs [36]. Until now, however, little was known about utilizing PAH-degrading endophytic bacteria to degrade PAHs inside plants [37]. In the present study, a phenanthrene-degrading endophytic bacterium, *Massilia* sp. Pn2, was isolated from *Alopecurus aequalis* Sobol grown in PAH-contaminated soil. Strain Pn2 could degrade more than 95.0% of the phenanthrene (150 mg·L^−1^) within 48 hours in a shake-flask culture under environmental conditions. Furthermore, it could also degrade naphthalene, acenaphthene, anthracene and pyrene, showing the potential for degrading various PAHs. These results indicated that *Alopecurus aequalis* Sobol harbored an endophytic bacterium that could degrade PAHs quickly and efficiently. Additionally, this strain could re-colonize the root surface of plant, invade its internal tissues and degrade PAH compounds inside the plant, showing potential for reducing plant PAH contamination risk in polluted environments.

Bacteria with the ability to degrade phenanthrene have previously been reported, such as *Arthrobacter sulfureus, Acidovorax delafieldii* and *Brevibacterium* sp. [38], *Burkholderia* sp. [39], *Pseudomonas* sp. [40], *Janibacter* sp. [41], *Mycobacterium* sp. [42], and *Staphylococcus* sp. [43]. An isolate from *Massilia* sp. was first reported to be able to grow on an MSM plate with phenanthrene as the sole carbon source by Bodour *et al*., indicating that strains from *Massilia* sp. had the ability to degrade PAHs [44]. Recently, with PCR-DGGE technology, Zhang *et al.* [45] found that *Massilia* spp. always made up one of the dominant populations in PAH-contaminated soils with different PAH concentrations. It was then hypothesized that varying PAH concentrations may provide additional metabolic niches for members of the *Massilia* spp., thereby facilitating ecotype speciation. In this work, an efficient phenanthrene-degrading bacterium in the genus of *Massilia* sp. was isolated from the internal tissues of plants, and its ability to degrade PAHs was verified.

The successful use of endophytic bacteria to reduce plant organic contamination requires the presence of high counts of contaminant-degrading bacteria inside the plant tissues [46]. In general, endophytic bacteria can easily colonize target plants and then distribute themselves among the plant tissues [47]. In this study, strain Pn2 could colonize ryegrass plants via root inoculation, and it showed good survival in plant tissues during the experimental period. After 12 days of ryegrass growth, the cell counts of strain Pn2 in the ryegrass were all more than 10^4^ in different tissues and treatments. Therefore, high counts of phenanthrene-degrading bacteria in plants could continuously degrade phenanthrene inside plant tissues, reducing the plant phenanthrene contamination risk. Additionally, most of the Pn2 cells colonized the root surfaces, and some were released into the solution. These bacterial cells could degrade phenanthrene in solution and thus indirectly reduce plant uptake and the accumulation of phenanthrene.

The colonization of ryegrass by strain Pn2 significantly promoted ryegrass growth by increasing the fresh weight and dry weight, as well as ryegrass height and root length. Endophytic bacteria could primarily promote plant growth in three ways [48]: (1) by increasing the availability of growth-limiting elements, for example, by fixing nitrogen and solubilizing mineral nutrients that are unavailable to plants such as P and Fe [17]; (2) by producing phytohormones such as auxins, cytokinins and gibberellins [49], [50]; or (3) by exerting ACC deaminase activity and thus decreasing stress-induced ethylene [51], [52]. Some bacteria also can indirectly benefit plant growth by competing with pathogens and reducing their activity [53]. In polluted environments, many endophytes can assist their host plants in overcoming contaminant-induced stress responses, thus providing improved plant growth [54]. Furthermore, the metabolites of phenanthrene produced by strain Pn2 could also be used as nutrients to support plant growth.

In addition to degrading phenanthrene in plant tissues and promoting plant growth, as mentioned above, additional mechanisms involved in the reduction of plant phenanthrene contamination risk by endophytes is the promotion of metabolic gene expression and the regulation of the plant's enzyme system activity [55], [56]. For instance, when contaminated with petroleum hydrocarbon, the prevalence of pollutant catabolic genes, such as alkane monooxygenase (*alk*B) and naphthalene dioxygenase (*ndo*B), was enhanced by 2- to 4-fold in the endophytes within plants [55]. Additionally, a series of other enzymes, such as esterase, oxidase, reductase, and dehalogenase, directly participates in the metabolism of anthropogenic contaminants inside plants [57]. Endophytic bacteria can affect the activities of these enzymes and thus regulate the metabolic processes of organic contaminants in plants. For example, when exposed to 10 mg·L^−1^ of trichloroethylene (TCE), the activities of catalase and superoxide dismutase significantly increased, whereas after incubation with the endophytic strain *Burkholderia cepacia* VM1468, the enzyme activities in the plants decreased [58].

## Conclusions

The presence of endophytic bacteria with the capacity to degrade PAHs in plants has important implications for growing plants in PAH-contaminated environments. In this study, an effective phenanthrene-degrading endophytic bacterium, *Massilia* sp. Pn2, was isolated from *Alopecurus aequalis* Sobol grown in PAH-contaminated soil. Pn2 could degrade more than 95% phenanthrene (150 mg·L^−1^) in MSM with phenanthrene as the sole carbon source within 48 hours under environmental conditions. Additionally, Pn2 could colonize plant tissues and efficiently reduce the risk of plant phenanthrene contamination. Although the precise interactions between the endophyte and the plant in phenanthrene degradation require further exploration, the potential use of strain Pn2 for reducing plant phenanthrene contamination risk is strongly supported by the research outlined here. We have provided new perspectives on the control of the plant uptake and accumulation of phenanthrene by endophytic bacteria. Furthermore, because strain Pn2 can degrade several PAHs, it represents a novel and useful bacterial resource for eliminating plant PAH contamination in polluted environments.
